# Imaging Modalities to Delineate Sequelae of Spontaneous Coronary Artery Dissection Managed with Percutaneous Coronary Intervention

**DOI:** 10.7759/cureus.7591

**Published:** 2020-04-08

**Authors:** Talha Ahmed, Jean Jeudy, Mukta C Srivastava

**Affiliations:** 1 Internal Medicine, University of Maryland Medical Center, Baltimore, USA; 2 Radiology, University of Maryland Medical Center, Baltimore, USA; 3 Interventional Cardiology, University of Maryland Medical Center, Baltimore, USA

**Keywords:** spontaneous coronary artery dissection, management, percutaneous coronary intervention, intravascular imaging, non-invasive imaging modalities

## Abstract

Patients with spontaneous coronary artery dissection (SCAD) often present as clinically unstable, requiring management with percutaneous coronary intervention. Complications from persistent dissection and initial percutaneous management can present with later challenges. Intravascular and non-invasive imaging modalities are adjunct modalities to coronary angiography to delineate coronary anatomy and guide therapeutic strategies. Herein, we describe the case of a 23-year-old female patient who presented with heart failure and severe mitral regurgitation eight months after an index SCAD event during which she underwent extensive stenting of the left anterior descending artery. The multimodal imaging used in this case and how it helped in formulating the management rationale is also reviewed.

## Introduction

We describe the case of a young woman who presented with heart failure from ventricular failure and severe mitral regurgitation eight months after an index spontaneous coronary artery dissection (SCAD) event during which she underwent extensive stenting of the left anterior descending artery (LAD). Coronary angiography (CA) performed as part of the pre-operative workup for mitral valve repair demonstrated areas of residual dissection as well as in-stent re-stenosis demonstrating long-term complications and management challenges of SCAD. The utility of complimentary imaging modalities such as CA, optic coherence tomography (OCT), and coronary computed tomography angiography (CCTA) in characterizing the complex anatomy of persistent dissection in SCAD and in guiding the management challenges in this scenario is discussed [1].

## Case presentation

**Figure 1:**
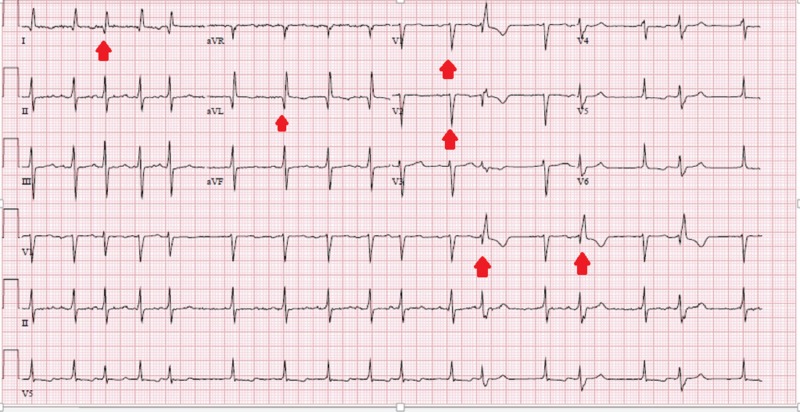
Electrocardiogram showing occasional premature ventricular complexes, Q-waves in leads I, avL, V1, and V2 suggesting a prior antero-lateral infarct, and irregular rhythm suggesting atrial fibrillation (red arrows)

A 23-year-old female presented with symptoms of chest pain and heart failure. Her history was significant for SCAD of the LAD eight months ago during pregnancy, which was managed by bare-metal stents to the mid-LAD. Vitals signs showed a heart rate of 90 beats per minute and a blood pressure of 110/65 mm Hg, and the patient was afebrile with normal oxygen saturation on room air. Her physical examination showed bi-basilar rales and an apical holosystolic murmur. There was no jugular venous distention or pedal edema on examination. Differential diagnosis included recurrent SCAD, development of an ischemic cardiomyopathy related to her SCAD event, and manifestation of a peri-partum cardiomyopathy. Electrocardiogram showed new-onset atrial fibrillation, occasional premature ventricular complexes, and prior antero-lateral infarct (Figure 1).

**Figure 2:**
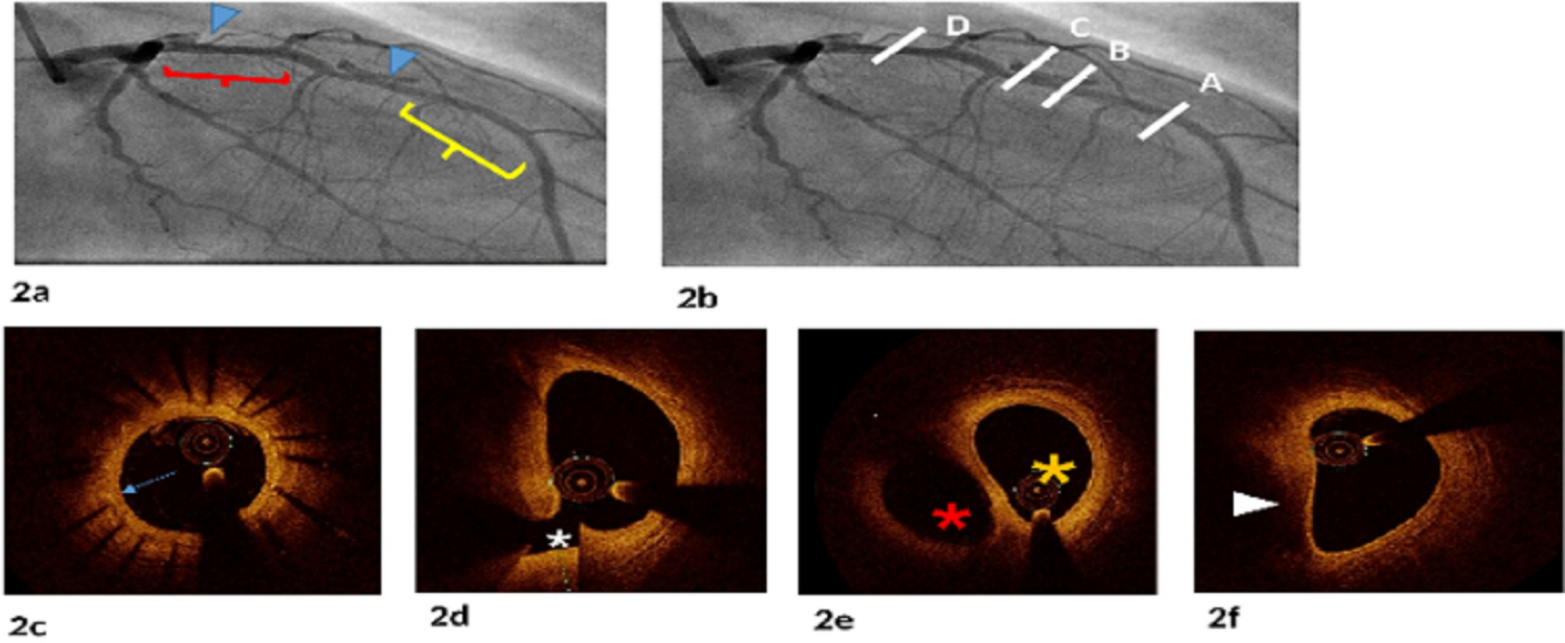
Coronary angiography with the corresponding OCT cross-sections (2a) Coronary angiography demonstrating mid-LAD and proximal-LAD areas of extravascular staining (blue arrowheads), area of mid-LAD stenting with diffuse mild in-stent re-stenosis (yellow bracket), and diffuse proximal LAD narrowing consistent with compression from the false lumen (red bracket). (2b) Coronary angiography with white hashes A–D corresponding to OCT cross-sections in 2c–2f. (2c) OCT image corresponding to cross-section A in 2b, showing diffuse concentric mild intimal thickening (blue arrow) within mid-LAD stents. (2d) OCT image corresponding to cross-section B in 2b, showing intimal flap (white asterisk) with initiation of communication of the true and false lumens. (2e) OCT image corresponding to cross-section C in 2b, showing the true lumen (yellow asterisk) with intima devoid of intimal plaque and patent false lumen (red asterisk). (2f) OCT image corresponding to cross-section D in 2b, showing compression of the proximal LAD by the thrombosed false lumen (white arrowhead). OCT, optical coherence tomography; LAD, left anterior descending artery

Echocardiography showed bi-ventricular dysfunction (either from a sequalae of untreated index SCAD or from peri-partum cardiomyopathy or atrial fibrillation induced cardiomyopathy). There was left ventricular (LV) dilatation with global hypokinesis, estimated LV ejection fraction of 25%, and significant functional mitral and tricuspid regurgitation. Guideline-directed medical therapy for heart failure was initiated. After no significant improvement over several weeks, she was recommended for surgical valve repair. Pre-operative CA demonstrated LAD stents with mild in-stent re-stenosis, two areas of extra-luminal dye staining, one at the proximal edge of the stents and one at a second un-stented proximal LAD site, and diffuse narrowing of the proximal un-stented LAD. Intravascular imaging with OCT was performed for further anatomic delineation, which confirmed mild in-stent re-stenosis and demonstrated an intimal flap with communication between a true lumen and a false lumen at the proximal stent edge, corresponding with the area of extravascular dye staining noted by CA, true and false lumens with normal true lumen intima, and compression of the proximal LAD by a thrombosed false lumen (Figure 2).

**Figure 3:**
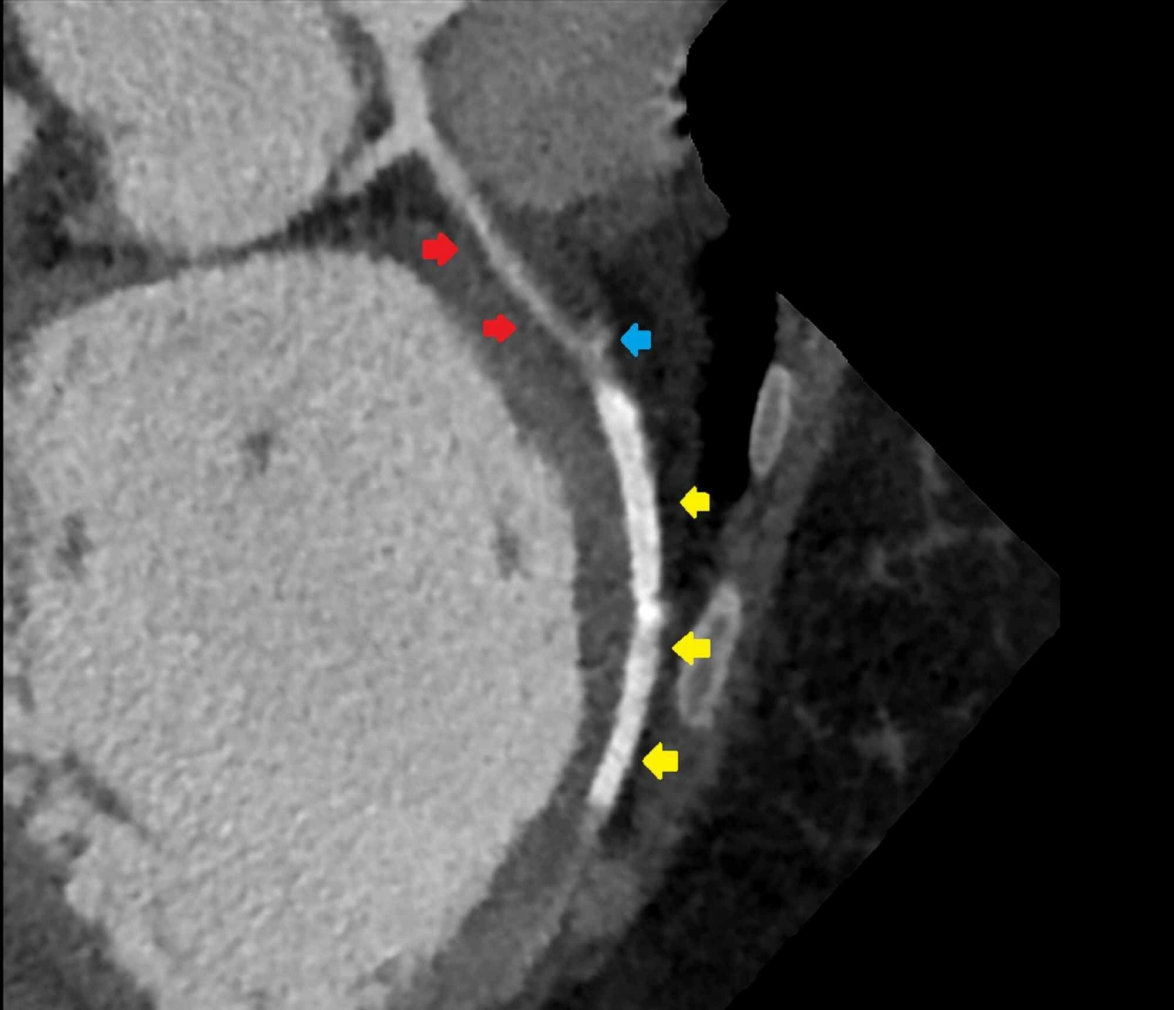
Coronary computed tomography angiography showing proximity of the thrombosed false lumen of dissection to origin of the left main artery (red arrows), proximal left anterior descending artery area of true and false lumen communication (blue arrow), and mid-left anterior descending artery stent (yellow arrows)

CCTA corroborated a segment of the thrombosed false lumen compressing the proximal LAD and the outpouchings of contrast in the proximal LAD and at the proximal stent edge to be consistent with residual communication of the true lumen with the false lumen. Additionally, CCTA precisely localized residual dissection in the proximal LAD to end within 10 mm of the left main (LM) artery bifurcation (Figure 3).

**Figure 4:**
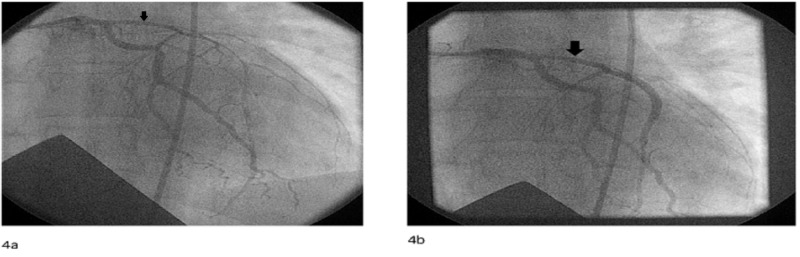
Coronary angiography at the index presentation showing residual dissection after stenting of the left anterior descending artery (4a) Diffuse left anterior descending artery dissection at index presentation (black arrow). (4b) Diffuse residual proximal left anterior descending artery narrowing suggestive of residual uncovered dissection after stenting at index presentation (black arrow).

Initial suspicion was that our patient had residual dissection from her initial event as index angiography demonstrated proximal LAD narrowing at case-end, suspicious for persistent luminal compression from a false lumen (Figure 4).

Management considerations included repeat LAD PCI with drug-eluting stents (DESs) with adjunctive intravascular imaging to ensure sealing of residual dissection, surgical LAD revascularization during valve surgery, and no further coronary intervention. A strategy of grafting the LAD during mitral and tricuspid valve repair was pursued. Screening for fibromuscular disease was not performed as the SCAD was attributed to be related to pregnancy and its complication.

## Discussion

SCAD is an increasingly recognized acute syndrome with non-atherosclerotic pathology, predominantly affecting women, characterized by spontaneous hemorrhage of medial vessels with intramural hematoma (IMH) formation, leading to coronary lumen compression and secondary intimal tears. In pregnancy, hormonally driven alteration of coronary wall architecture can predispose to SCAD [2].

CA is the mainstay of diagnosis; however, dissections without intimal tears may manifest as diffuse smooth vessel luminal narrowing, requiring high suspicion or intravascular imaging with intravascular ultrasound (IVUS) or OCT for diagnostic confirmation. When PCI is entertained or when diagnosis is unclear, intravascular imaging, if possible, can demonstrate intimal tears, IMHs, and areas compressed by the false lumen, as well as confirm the absence of atherosclerotic plaque in the intima of the true lumen to support a SCAD diagnosis. Due to higher resolution imaging, OCT is possibly a superior modality for SCAD delineation at the expense of increased dye use and lesser depth penetration than IVUS [3]. There is, however, an inherent risk of these diagnostic intravascular procedures (OCT and IVUS). This includes propagation of the dissection, and hence careful consideration should be given when evaluating which patients will benefit from these procedures.

Supportive treatment is the preferred management strategy for SCAD and carries a good prognosis for resolution; however, ongoing infarction and instability can require emergent revascularization with PCI or coronary artery bypass grafting (CABG). Acute revascularization in SCAD with PCI is plagued by high failure rates through several mechanisms such as dissection extension from vessel manipulation, IMH displacement during stent deployment, and stent edge dissection from stent deployment in a vessel segment with ongoing dissection [4]. Due to the need for long stents with clinical scenarios frequently mandating bare-metal stent selection, re-stenosis risk is high. Late stent malapposition can result once IMHs reabsorb, predisposing to both re-stenosis and stent thrombosis [5,6]. Notably, in a contemporary series including 750 SCAD patients, only 29% of patients treated with PCI were considered to have complete acute procedural success [7].

We suspect that our patient had residual dissection from her initial event as index angiography demonstrated proximal LAD narrowing at case-end, suspicious for persistent luminal compression from a false lumen. The intimal flap at the proximal stent edge suggests an edge dissection sustained when the stent was deployed in an area of propagating false lumen. The index event did not occur at our institution; however, we theorize that patient in-stability precluded intravascular imaging prior to stenting. Nevertheless, we would maintain that at case-end, such imaging would have had utility in optimizing the PCI result [8,9].

Complexities of the anatomy elaborated by the imaging modalities used, including dissection proximity to the LM coronary artery, multiple sites of communication between the true and false lumens, and evidence of early diffuse in-stent re-stenosis, further made PCI an unfavorable approach. No further intervention was deemed a precarious option given the concern for dissection expansion into the LM coronary artery. Because the management plan included valve surgery, CABG was considered the most favorable revascularization option, with careful intravascular imaging-guided PCI with DES the next preferred option in case the patient not undergoing surgery.

## Conclusions

We conclude that in PCI-managed SCAD, intra-vascular imaging for intervention optimization should be performed when feasible, and in conjunction with CA, it provides complete evaluation of the SCAD lesion. If invasive evaluation does not fully elucidate SCAD anatomy, CCTA can have added utility, providing a non-invasive modality for follow-up imaging post-PCI. Revascularization options can be limited in patients with persistent complex dissection post-PCI and must be evaluated in the context of clinical and anatomical features.
